# Protective effects of imeglimin on the development of atherosclerosis in ApoE KO mice treated with STZ

**DOI:** 10.1186/s12933-024-02189-z

**Published:** 2024-03-19

**Authors:** Junpei Sanada, Tomohiko Kimura, Masashi Shimoda, Yuichiro Iwamoto, Hideyuki Iwamoto, Kazunori Dan, Yoshiro Fushimi, Yukino Katakura, Yuka Nogami, Yoshiko Shirakiya, Yuki Yamasaki, Tomoko Ikeda, Shuhei Nakanishi, Tomoatsu Mune, Kohei Kaku, Hideaki Kaneto

**Affiliations:** Department of Diabetes, Endocrinology and Metabolism, Kawasaki Medical School, 577 Matsushima, Kurashiki, 701-0192 Japan

**Keywords:** Imeglimin, Type 2 diabetes mellitus, Atherosclerosis, Oxidative stress, Inflammation

## Abstract

**Background:**

Imeglimin is a new anti-diabetic drug which promotes insulin secretion from pancreatic β-cells and reduces insulin resistance in insulin target tissues. However, there have been no reports examining the possible anti-atherosclerotic effects of imeglimin. In this study, we investigated the possible anti-atherosclerotic effects of imeglimin using atherosclerosis model ApoE KO mice treated with streptozotocin (STZ).

**Methods:**

ApoE KO mice were divided into three groups: the first group was a normoglycemic group without injecting STZ (non-DM group, n = 10). In the second group, mice were injected with STZ and treated with 0.5% carboxymethyl cellulose (CMC) (control group, n = 12). In the third group, mice were injected with STZ and treated with imeglimin (200 mg/kg, twice daily oral gavage, n = 12). We observed the mice in the three groups from 10 to 18 weeks of age. Plaque formation in aortic arch and expression levels of various vascular factors in abdominal aorta were evaluated for each group.

**Results:**

Imeglimin showed favorable effects on the development of plaque formation in the aortic arch in STZ-induced hyperglycemic ApoE KO mice which was independent of glycemic and lipid control. Migration and proliferation of vascular smooth muscle cells and infiltration of macrophage were observed in atherosclerotic lesions in STZ-induced hyperglycemic ApoE KO mice, however, which were markedly reduced by imeglimin treatment. In addition, imeglimin reduced oxidative stress, inflammation and inflammasome in hyperglycemic ApoE KO mice. Expression levels of macrophage makers were also significantly reduced by imeglimin treatment.

**Conclusions:**

Imeglimin exerts favorable effects on the development of plaque formation and progression of atherosclerosis.

**Supplementary Information:**

The online version contains supplementary material available at 10.1186/s12933-024-02189-z.

## Background

Atherosclerosis is caused by vascular wall thickening, which mainly consists of proliferation and degeneration of vascular walls. Myocardial infarction and stroke develop with arteriosclerosis as an underlying disease, and the incidence is higher in diabetic patients than in non-diabetic patients [1]. Management of risk factors such as dyslipidemia, hypertension, diabetes and smoking is important to prevent the progression of atherosclerosis. It has been reported that anti-diabetic drugs inhibit the progression of atherosclerosis. It is thought that anti-diabetic drugs exert anti-atherosclerotic effects through amelioration of blood vessel inflammation by improving glycemic control, vasodilation by activation of nitric oxide (NO), suppression of platelet aggregation, and suppression of expression of cell adhesion factors [2–7]. Several large-scale double-blind, randomized placebo-controlled trials have reported that GLP-1 receptor agonists and SGLT2 inhibitor such as liraglutide (LEADER trial), semaglutide (SUSTAIN-6 trial), albiglutide (Harmony Outcomes), oral semaglutide (PIONEER trial), dulaglutide (REWIND trial), empagliflozin (EMPA-REG OUTCOME) and canagliflozin (CANVAS Program) reduced 3-point major adverse cardiovascular events [8–14].

Imeglimin is an anti-diabetic drug that promotes insulin secretion [15, 16] and reduces insulin resistance [17, 18]. It has been reported that imeglimin has the effect of protecting β-cells by improving endoplasmic reticulum stress, oxidative stress and inflammation [19–21]. Improving inflammation and stress markers may occur not only in pancreatic β-cells but also in vascular endothelium. There have been several reports about the possible effects of imeglimin on endothelial dysfunction [22, 23]. For example, it was reported that imeglimin treatment improved endothelial function in subjects with type 2 diabetes by enhancing postprandial blood flow-mediated dilation. It was also reported that imeglimin improved cardiac and kidney function in experimental animals by improving mitochondrial and endothelial function. There have been no reports, however, that examined the anti-atherosclerotic effects of imeglimin. We hypothesized that imeglimin reduces vascular endothelial dysfunction by reducing oxidative stress and thus exerts some favorable anti-atherosclerotic effects. In this study, we investigated the anti-atherosclerotic effects of imeglimin using arteriosclerosis model mice. To the best of our knowledge, this is the first report showing anti-atherosclerotic effects of imeglimin.

## Methods

### Animals and diets

ApoE KO mice (C57BL/6J-ApoEtm1Unc) were purchased from Charles River Laboratories and housed (two animals per cage for all experiments) under controlled ambient conditions and 12 h light and dark cycle. The animals were given free access to water and standard diet (MF; Oriental Yeast Co., Ltd.) and maintained at 25 °C.

We used streptozotocin (STZ) to induce hyperglycemia based on our previous report [7]. ApoE KO mice were intraperitoneally injected with STZ (FUJIFILM Wako Pure Chemical Corporation, Japan) (50 mg/kg/day) for 5 consecutive days at 8 weeks of age. Blood glucose levels were measured every other day at 9 weeks of age. When mice showed apparent hyperglycemia superior to 300 mg/dL at fed status more than twice continuously until 10 weeks of age, ApoE KO mice were divided into three groups: the first group was a normoglycemic group without injecting STZ (non-DM group, n = 10). In the second group, mice were injected with STZ and treated with 0.5% carboxymethyl cellulose (CMC) (control group, n = 12). In the third group, mice were injected with STZ and treated with imeglimin (200 mg/kg, twice daily oral gavage, purchased from Sumitomo Pharma) as we previously reported (imeglimin group, n = 12) [20]. We observed the mice in the three groups from 10 to 18 weeks of age. Body weight and food intake were measured during the experiment period.

The study was approved by the animal use committee of Kawasaki Medical School (No. 23-001) and conducted in compliance with the animal use guidelines of Kawasaki Medical School.

### Measurement of biochemical markers

Blood samples were collected from tail vein. Blood glucose levels were measured using a glucometer (Glutest Mint; Sanwa Kagaku Kenkyusho Co, Ltd, Japan). Blood glucose levels were measured with one sample for each measurement in principle, because it was known that the accuracy of glucometer we used was quite high. However, when blood glucose levels were obviously strange due to some reason such as insufficient blood collection, we measured blood glucose levels again and confirmed the accuracy of the levels. Plasma total cholesterol, triglyceride, LDL-cholesterol and HDL-cholesterol were measured enzymatically using the Wako LabAssay, L type Wako (Wako Pure Chemical Industries, Japan). Urine was collected using metabolic cage at 18 weeks of age, and urinary 8-OHdG levels were measured using ELISA kit (Japan Institute for the Control of Aging, NIKKEN SEIL Co, Ltd, Japan).

### RNA isolation and real time PCR

Inflammation and macrophage makers were assessed using mRNA extracted from the abdominal aorta. Total RNA extraction was performed using a RNeasy lipid tissue mini kit (QIAGEN, Valencia, CA) according to the manufacturers’ instructions. cDNA was produced from mRNA using TaqMan reverse transcription reagents (Applied Biosystems, Foster City, CA). Quantitative RT-PCR was conducted using a Step One Plus Real-Time PCR system (Applied Biosystems). To quantify gene expression, the 2^−ΔCT^ was calculated using β-actin as an internal control. Primer sequences used for real time PCR are presented in Table 1.

**Table 1:** Primer sequences of forward and reverse primers for real-time PCR

Genes	Forward	Reverse
β-actin	CGTGAAAAGATGACCCAGATCA	CACAGCCTGGATGGCTACGTA
NLRP3	CCTTGGACCAGGTTCAGTGT	AGGCAGCAGTTCACCAGTCT
IL-1β	TGGTGTGTGACGTTCCCATTA	CGACAGCACGAGGCTTTTTT
MCP-1	CTTCCTCCACCACCATGCA	CCAGCCGGCAACTGTGA
iNOS	GTGACGGCAAACATGACTTCA	GCCATCGGGCATCTGGTA
VCAM-1	GATCTCCCCTGAATACAAAACGAT	GCCCGTAGTGCTGCAAGTG
ICAM-1	TCGGAAGGGAGCCAAGTAACT	CGACGCCGCTCAGAAGAA
F4/80	TGCATCTAGCAATGGACAGC	GCCTTCTGGATCCATTTGAA
CD68	TTTCTCCAGCTGTTCACCTTGA	CCCGAAGTGTCCCTTGTCA
TIMP-1	GCATGGACATTTATTCTCCACTGT	TCTCTAGGAGCCCCGATCTG
MMP-2	CCCTCAAGAAGATGCAGAAGTTC	TCTTGGCTTCCGCATGGT

### Histological and immunehistological analyses

Under anesthesia, PBS was perfused from left ventricle and then animals were killed and heart and aorta were dissected. Sudan IV (Wako: 192-04392) staining was conducted for aortic arch. Adventitial fat tissue was removed and aorta was dissected longitudinally. The image analysis software NIH Image (version 1.61; http://rsbweb.nih.gov/ij/) was used to calculate the ratio of the plaque lesion to the total aortic arch area.

Isolated thoracic aorta was fixed overnight with formalin at 4 °C. Tissue was routinely processed for paraffin embedding and 4 μm sections of thoracic aorta were cut and mounted on silanized slides and were stained by Hematoxylin Eosin (HE), alpha-smooth muscle actin (α-SMA) (NB300-978, RRID:AB_2273628) and CD68 (ab125212, RRID:AB_10975465, 1:500).

### Statistical analysis

All data were analyzed and expressed as the mean ± standard error of the mean. Differences between two groups were tested for statistical significance using Student’s t-test. p values less than 0.05 were considered to indicate a statistically significant difference.

## Results

### There was no significant difference between control group and imeglimin group in blood glucose levels, body weights and lipid-related markers

**Fig. 1:**
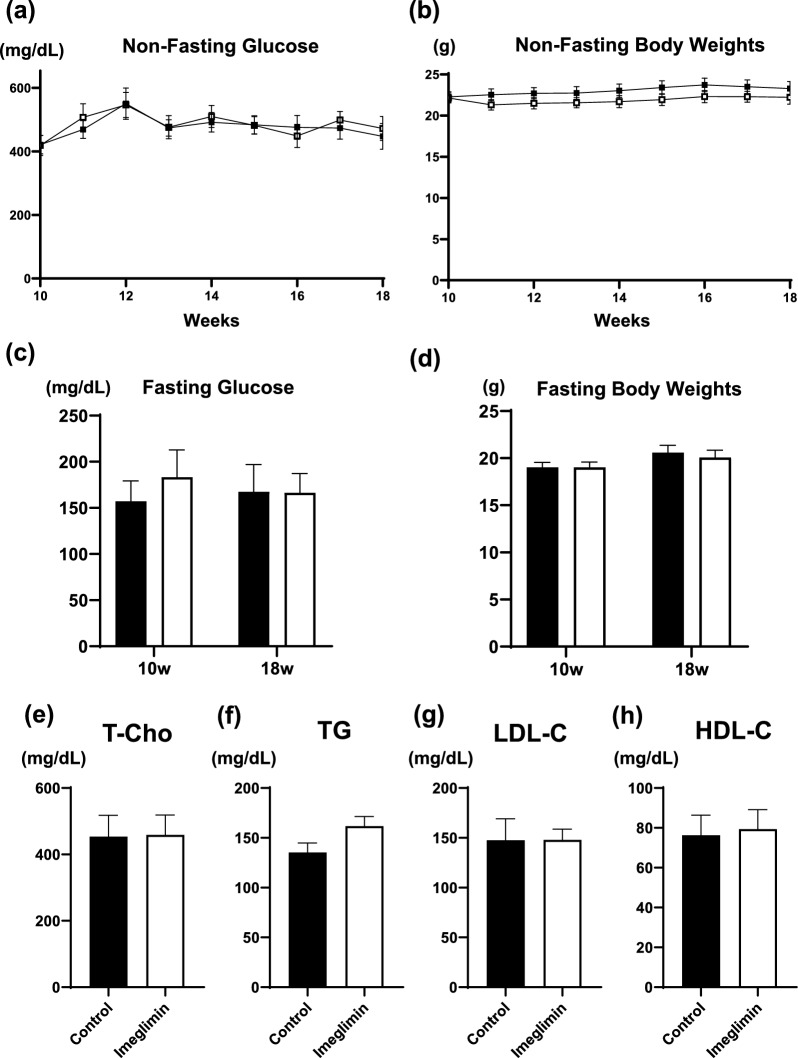
**a**–**d** There was no significant difference between untreated DM mice (control group) and imeglimin-treated DM mice (imeglimin group) in non-fasting and fasting blood glucose levels and body weights. **e**–**h** Total cholesterol, triglyceride, HDL-cholesterol and LDL-cholesterol levels were not significantly different between the two groups. Black squares and bars, untreated DM mice (control group); White squares and bars, imeglimin-treated DM mice (imeglimin group). n = 9–12

It is well known that glycemic and lipid control greatly affect the progression of atherosclerosis. Therefore, we evaluated glycemic and lipid control in ApoE KO mice with and without imeglimin treatment. Between 10 and 18 weeks of age, we measured non-fasting blood glucose levels and body weights every week in ApoE KO mice with and without imeglimin treatment. There was no significant difference between untreated DM mice (control group) and imeglimin-treated DM mice (imeglimin group) in non-fasting blood glucose levels and body weights (Fig. 1a, b). At the start and end of intervention, there was no difference in fasting blood glucose levels and fasting body weights between the two groups (Fig. 1c, d). Next, we measured lipid levels at the end of intervention. Total cholesterol, triglyceride, HDL-cholesterol and LDL-cholesterol levels were not significantly different between the two groups (Fig. 1e–h). In addition, food intake and organ weights (liver, brown adipose tissue, muscle, kidney, pancreas and heart) were not different between the two groups (Additional file 1: Figure S1a, b). These data indicate that the amount of imeglimin used in this study does not influence glycemic and lipid control in ApoE KO mice. The mortality was 0% in each group during the observation period in this study.

### Plaque formation in aortic arch was significantly reduced by imeglimin treatment

**Fig. 2:**
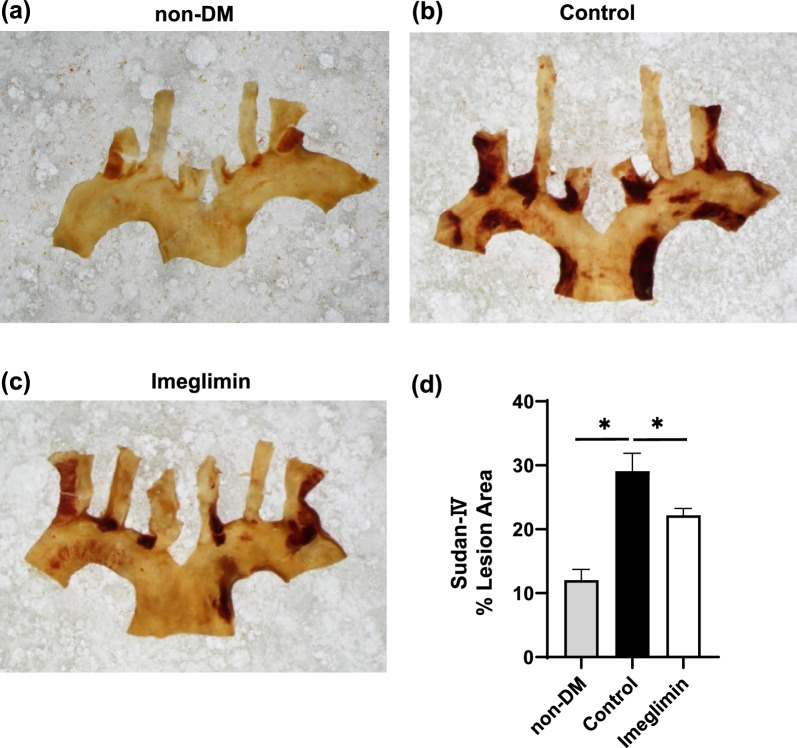
**a**, **b**, **d** In the aortic aorta in untreated DM mice (control group), significantly increased plaque formation was observed compared to non-DM group. **c**, **d** In imeglimin-treated group, plaque development was significantly reduced compared to control group. n = 8. *: p < 0.05

To investigate anti-atherogenic effect of imeglimin, we evaluated plaque formation in the aortic arch. Normoglycemic mice that were not treated with STZ (non-DM group) had minimal plaque development in the aortic arch (Fig. 2a). In the aortic aorta in untreated DM mice (control group), significantly increased plaque formation was observed compared to non-DM group (Fig. 2b, c). In imeglimin-treated group, plaque development was significantly reduced compared to control group (Fig. 2d). These data indicate that imeglimin has favorable effects on the development of plaque formation in the aortic arch.

### Migration and proliferation of vascular smooth muscle cells and infiltration of macrophages were observed in atherosclerotic lesions but they were significantly reduced by imeglimin treatment

**Fig. 3:**
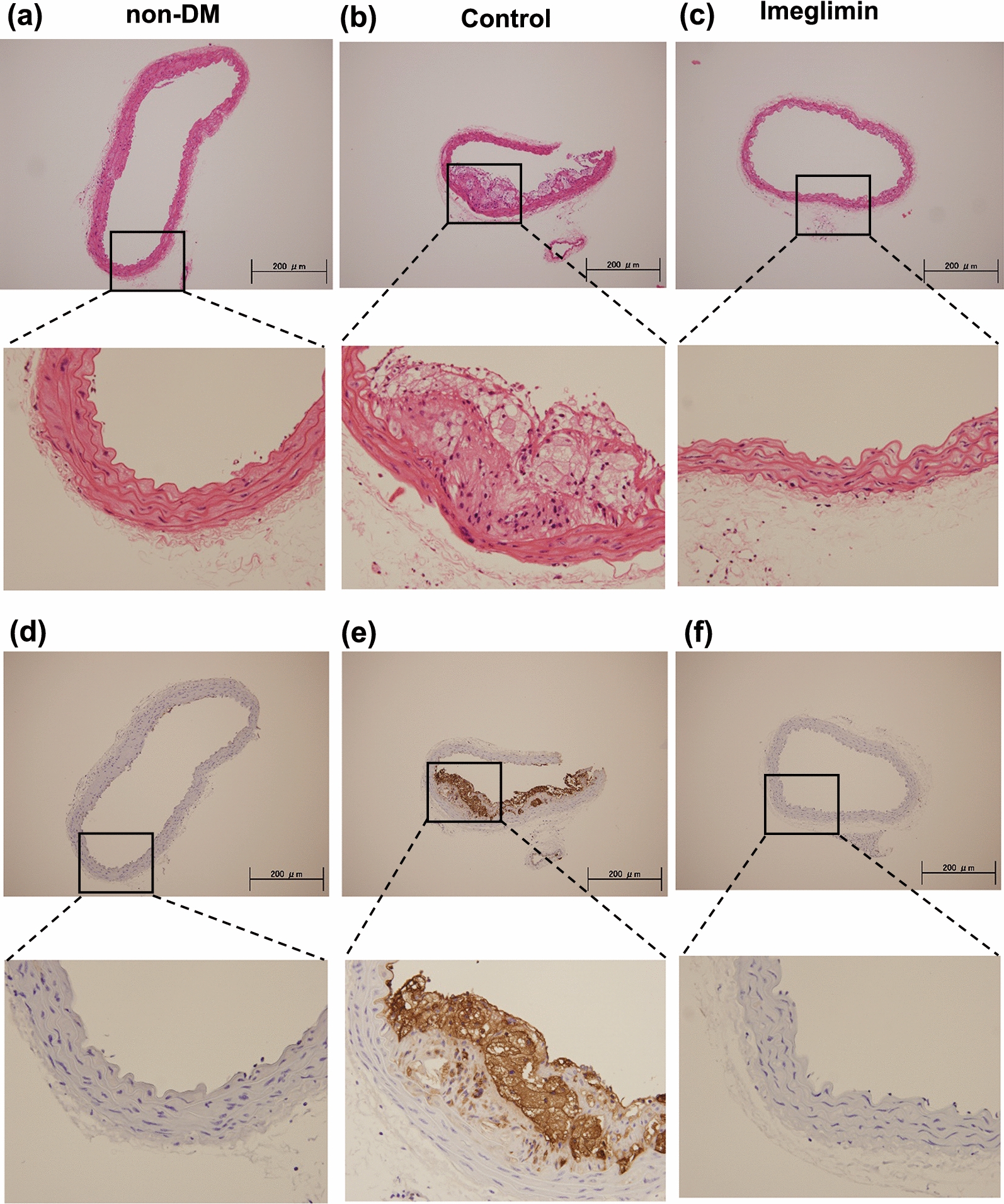
**a**–**c** In HE staining of the thoracic aorta, substantial plaque development was observed in untreated DM mice (control group) compared to non-DM mice. In imeglimin-treated DM mice, plaque development was markedly reduced compared to control group. **d**–**f** In CD68 staining of the aortic aorta, quite large CD68 positive area was observed in control group compared to non-DM group. In addition, CD68 positive area coincided with the plaque area. In imeglimin-treated DM mice, CD68 positive area was markedly reduced compared to control group

To examine the details of plaque formation, we performed HE staining and immunostaining of thoracic aortic wall. In HE staining of the thoracic aorta, substantial plaque development was observed in untreated DM mice (control group) compared to non-DM mice. In imeglimin-treated DM mice, plaque development was markedly reduced compared to control group (Fig. 3a–c). Next, to assess the vascular intima and media, we performed α-SMA staining. In α-SMA staining, proliferation and extension of vascular smooth muscle cells into the intima was observed in untreated DM mice (control group) compared to non-DM mice. In imeglimin-treated DM mice, plaque development was markedly reduced compared to control group (Additional file 1: Figure S2a–c). Additionally, to evaluate macrophage infiltration into the vessel wall, we performed CD68 staining. In CD68 staining of the aortic aorta, quite large CD68 positive area was observed in control group compared to non-DM group. In addition, CD68 positive area coincided with the plaque area. In imeglimin-treated DM mice, CD68 positive area was markedly reduced compared to control group (Fig. 3d–f).

### Imeglimin reduced oxidative stress and suppressed expression levels of inflammation and inflammasome factors in abdominal aorta

**Fig. 4:**
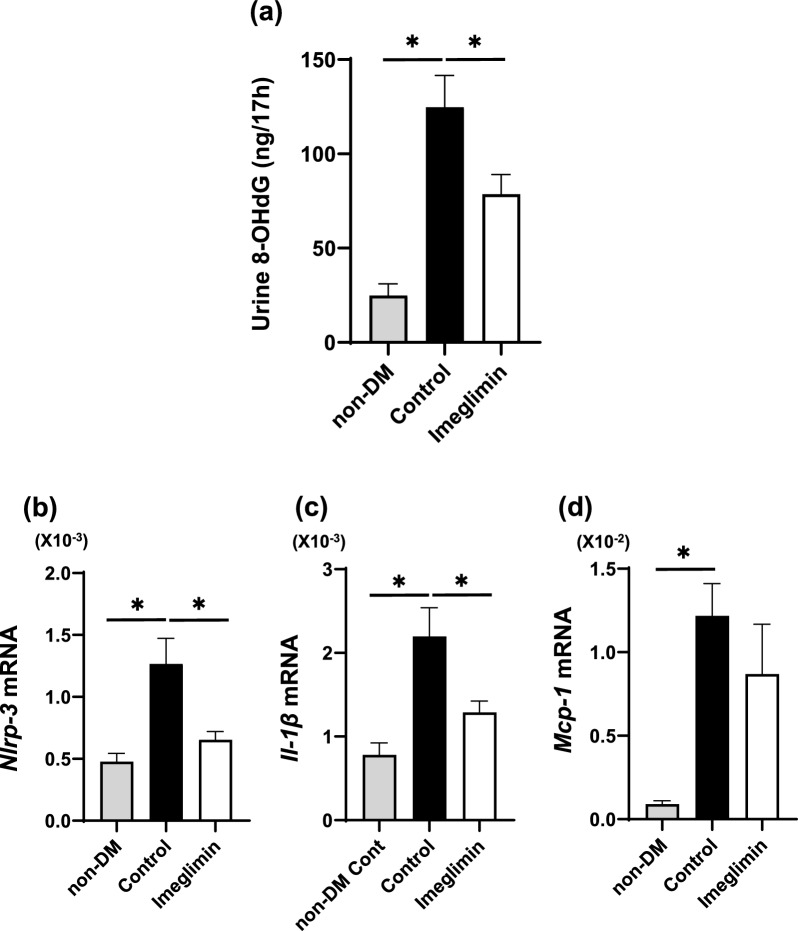
**a** Urinary 8-OHdG levels were significantly higher in untreated DM group (control group) compared to non-DM group. And in imeglimin-treated DM group, urinary 8-OHdG levels were significantly lower compared to untreated DM group (control group). n = 4–6. **b**–**d** Expression levels of *NLRP-3*, *IL-1β* and *MCP-1* mRNA were significantly higher in control group compared to non-DM group. *NLRP-3* and *IL-1β* levels were significantly lower in imeglimin group compared to control group. n = 8–10. *: p < 0.05

It has been reported that imeglimin reduces the production of reactive oxygen species (ROS) by competitively inhibiting mitochondrial respiratory chain complex 1. In addition, it is well known that increased production of ROS is involved in the progression of atherosclerosis. Therefore, we measured levels of urinary 8-OHdG, an oxidative stress marker, in our study. Urinary 8-OHdG levels were significantly higher in untreated DM group (control group) compared to non-DM group. And in imeglimin-treated DM group, urinary 8-OHdG levels were significantly lower compared to untreated DM group (control group) (Fig. 4a). It has been reported that increased oxidative stress induces inflammation. Therefore, we evaluated mRNA expression of inflammation and inflammasome factors in abdominal aorta. Expression levels of *NLRP-3*, *IL-1β* and *MCP-1* mRNA were significantly higher in untreated DM group (control group) compared to non-DM group (p < 0.05). *NLRP-3* and *IL-1β* levels were significantly lower in imeglimin group compared to control group (*p* < 0.05) (Fig. 4b–d). These results indicate that imeglimin reduces oxidative stress, inflammation and inflammasome in STZ-induced hyperglycemic ApoE KO mice.

### Expression levels of and macrophage makers were significantly reduced by imeglimin treatment

**Fig. 5:**
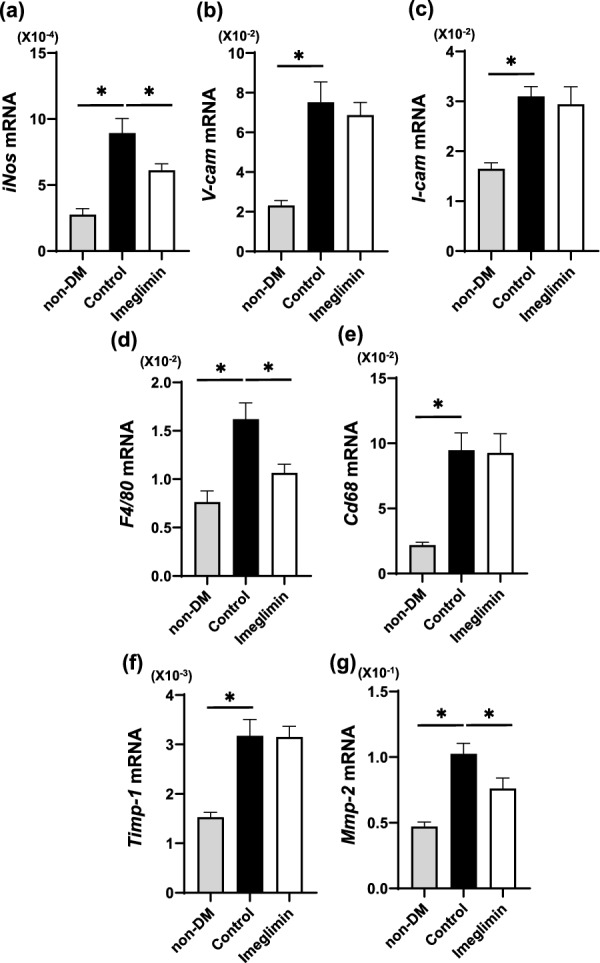
**a** Inducible NO synthase (*iNOS)* expression levels were significantly lower in imeglimin-treated mice compared to untreated DM mice (control group). **b**, **c** Expression levels of cell adhesion factors such as *Vcam-1* and *Icam-1* were not different between control and imeglimin group. **d** Expression level of a macrophage marker *F4/80* was significantly lower in imeglimin group compared to control group. **e** Expression level of *CD68* was not different between control and imeglimin group. **f**, **g** Concerning plaque stability markers *TIMP1* and *MMP2*, *TIMP1* levels were not different between control and imeglimin group, but *MMP2* level was significantly lower in imeglimin group compared to control group. n = 8–10. *: p < 0.05

To further examine the protective effects of imeglimin against atherosclerosis, we evaluated mRNA expression levels of various vascular factors. Inducible NO synthase (*iNOS)* expression levels were significantly lower in imeglimin-treated mice compared to untreated DM mice (control group) (*p* < 0.05) (Fig. 5a). Expression levels of cell adhesion factors such as *Vcam-1* and *Icam-1* were not different between control and imeglimin group (Fig. 5b, c). Expression level of a macrophage marker *F4/80* was significantly lower in imeglimin group compared to control group (*p* < 0.05) (Fig. 5d). Expression levels of *CD68* were not different between control and imeglimin group (Fig. 5e). Next, we evaluated expression levels of plaque stability markers *TIMP1* and *MMP2*. *TIMP1* level was not different between control and imeglimin group (Fig. 5f), but *MMP2* level was significantly lower in imeglimin group compared to control group (*p* < 0.05) (Fig. 5g).

## Discussions

In this study, we demonstrated that imeglimin showed beneficial effects on the development of plaque formation in the aorta in STZ-induced hyperglycemic ApoE KO mice, independently of glycemic and lipid control. In addition, imeglimin treatment significantly reduced migration and proliferation of vascular smooth muscle cells and infiltration of macrophages in atherosclerotic lesions. Furthermore, imeglimin treatment mitigated oxidative stress, inflammation and activation of inflammasome, which was presumably involved in the anti-atherosclerotic effects of imeglimin.

The mechanism underlying the development of atherosclerosis is generally believed to involve the following steps. First, endothelial cells are damaged by hypertension and chronic inflammation. Next, LDL-cholesterol enters the endothelium and oxidative LDL is formed. After oxidative LDL is processed by macrophage, it eventually forms a plaque. Therefore, in order to suppress the development of atherosclerosis, it is important to control hypertension, hyperglycemia, chronic inflammation due to increased oxidative stress, elevated LDL-cholesterol and activation of macrophages. In this study, imeglimin administration did not change blood glucose levels. Imeglimin is an antidiabetic drug, but there has been reported that it does not change blood glucose levels in mice [24]. Since the dosage of imeglimin in this study was based on previous reports [18, 20], we think that there was no problem with the dosage. There was no difference in lipid markers between control and imeglimin group. Blood pressure was not measured in this study, but we assume that imeglimin did not affect blood pressure because there has been no report at all showing anti-hypertensive effect of imeglimin. In this study, we think that reduction of ROS was involved in anti-atherosclerosis effect. The majority of ROS are produced in mitochondria, and ROS production increases when mitochondrial function is deteriorated such as under hyperglycemic conditions. ROS activate inflammation and inflammasome, which are involved in the development of atherosclerosis [25]. It has been reported that imeglimin acts on the mitochondrial respiratory chain complex and reduces the production of ROS [18]. We reported that chronic treatment with imeglimin reduced levels of urinary 8-OHdG, an oxidative stress marker, and reduced inflammation and apoptosis in islets in type 2 diabetic db/db mice [20]. In this study, levels of urinary 8-OHdG were significantly reduced by imeglimin treatment, and mRNA expressions of inflammation-related factors in abdominal aorta were significantly reduced.

In animal experiments with some rodents such as mice, when rodents are given free access to water and diet, fasting and/or non-fasting blood glucose levels are not necessarily decreased by several well-established anti-diabetic drugs which can exert glucose-lowering effects in human subjects with type 2 diabetes mellitus. Indeed, there is a report showing that imeglimin does not decrease fasting and/or non-fasting blood glucose levels in mouse model without strong glucose loading [24]. In addition, we ourselves had the following experience. Biguanide and sulfonylurea, both of which are often used as anti-diabetic drugs in everyday clinical practice, did not have glucose-lowering effects in rodents, at least in obese type 2 diabetic db/db mice, although we did not publish such data due to the lack of glucose-lowering effects. The precise reason remains unknown why some anti-diabetic drugs including imeglimin show glucose-lowering effects only in human subjects with type 2 diabetes mellitus but not in rodent diabetes models, but we assume that there are some differences in characteristics between human subjects and rodents. In addition, it was fortunate in some sense that blood glucose levels were not decreased by imeglimin treatment for the following reason. If blood glucose levels were decreased by imeglimin, it was difficult to judge whether imeglimin exerted protective effects on the development of atherosclerosis through a direct action of imeglimin or through the amelioration of glycemic control. Since blood glucose levels were not decreased by imeglimin in this study, we can confidently conclude that imeglimin has protective effects on the development of atherosclerosis through a direct action of imeglimin.

There is a limitation in this study. First, we think that administration of imeglimin suppressed the progression of atherosclerosis by improving mitochondrial function and reducing the production of ROS, but we failed to detect change in mitochondrial morphology or function after imeglimin treatment. Second, although this study showed that imeglimin decreased inflammation-related factor levels and suppressed macrophage infiltration in the aorta, further study would be important to fully unveil imeglimin action on macrophages. Finally, we started imeglimin treatment after inducing hyperglycemia at an early stage, but further study would be important to examine whether similar effects are obtained even at an advanced stage after the progression of atherosclerosis. We think that it is important to know from the clinical point of view in which timing imeglimin can exert most beneficial effects on atherosclerosis.

## Conclusions

Imeglimin exerts favorable effects on the development of plaque formation and progression of atherosclerosis. We think that the data in this manuscript would be very informative and useful not only for many clinicians but also many researchers in atherosclerosis as well as diabetes area. Also, we hope that the data in this manuscript would lead to even greater scientific progress in the future.

### Supplementary Information

**Additional file 1: Figure S1.** (a-b) There was no significant difference between untreated DM mice (control group) and imeglimin-treated DM mice (imeglimin group) in food intake and organ weights. Black squares and bars, untreated DM mice (control group); White squares and bars, imeglimin-treated DM mice. n = 9–12. **Figure S2.** (a)-(c) In α-SMA staining of the thoracic aorta, proliferation and extension of vascular smooth muscle cells into the intima was observed in untreated DM mice (control group) compared non-DM mice. In imeglimin-treated DM mice, plaque development was reduced compared to control group.

## Data Availability

The datasets generated and/or analyzed during the current study are available from the corresponding author upon reasonable request.

## Abbreviations

ApoE: Apolipoprotein E
STZ: Streptozotocin
TG: Triglyceride
LDL-C: Low density lipoprotein cholesterol
HDL-C: High density lipoprotein cholesterol
α-SMA: α-Smooth muscle actin
ROS: Reactive oxygen species
8-OHdG: 8-Hydroxy-2ʹ-deoxyguanosine
*Nlrp-3*: Nucleotide-binding oligomerization domain-like receptor family, pyrin domain-containing 3
*Il-1β*: Interleukin 1 β
*Mcp-1*: Monocyte chemotactic protein-1
*iNos*: Inducible nitric oxide synthase
*V-cam*: Vascular cell adhesion molecule
*I-cam*: Intracellular adhesion molecule
*Timp-1*: Tissue inhibitor metalloproteinase 1
*Mmp-2*: Matrix metalloproteinase 2
