# Supplementary figures and images for: Protective effects of imeglimin on the development of atherosclerosis in ApoE KO mice treated with STZ
Source: Cardiovasc Diabetol. 2024 Mar 19;23:105. doi: 10.1186/s12933-024-02189-z (PMC10953273; doi:10.1186/s12933-024-02189-z)

## Slide 1
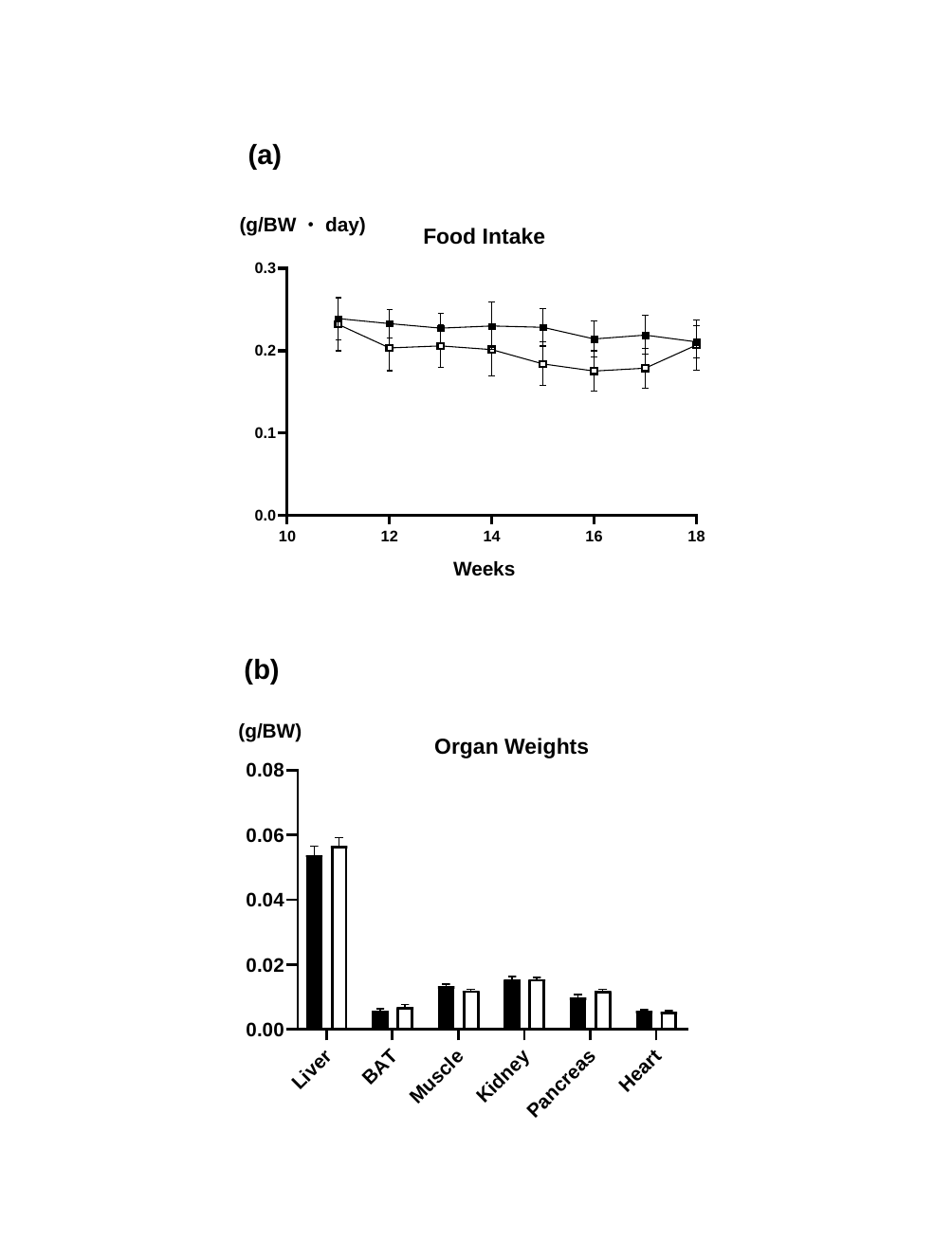

(a)
(g/BW・day)
Food Intake
Weeks
(b)
(g/BW)
Organ Weights

## Slide 2
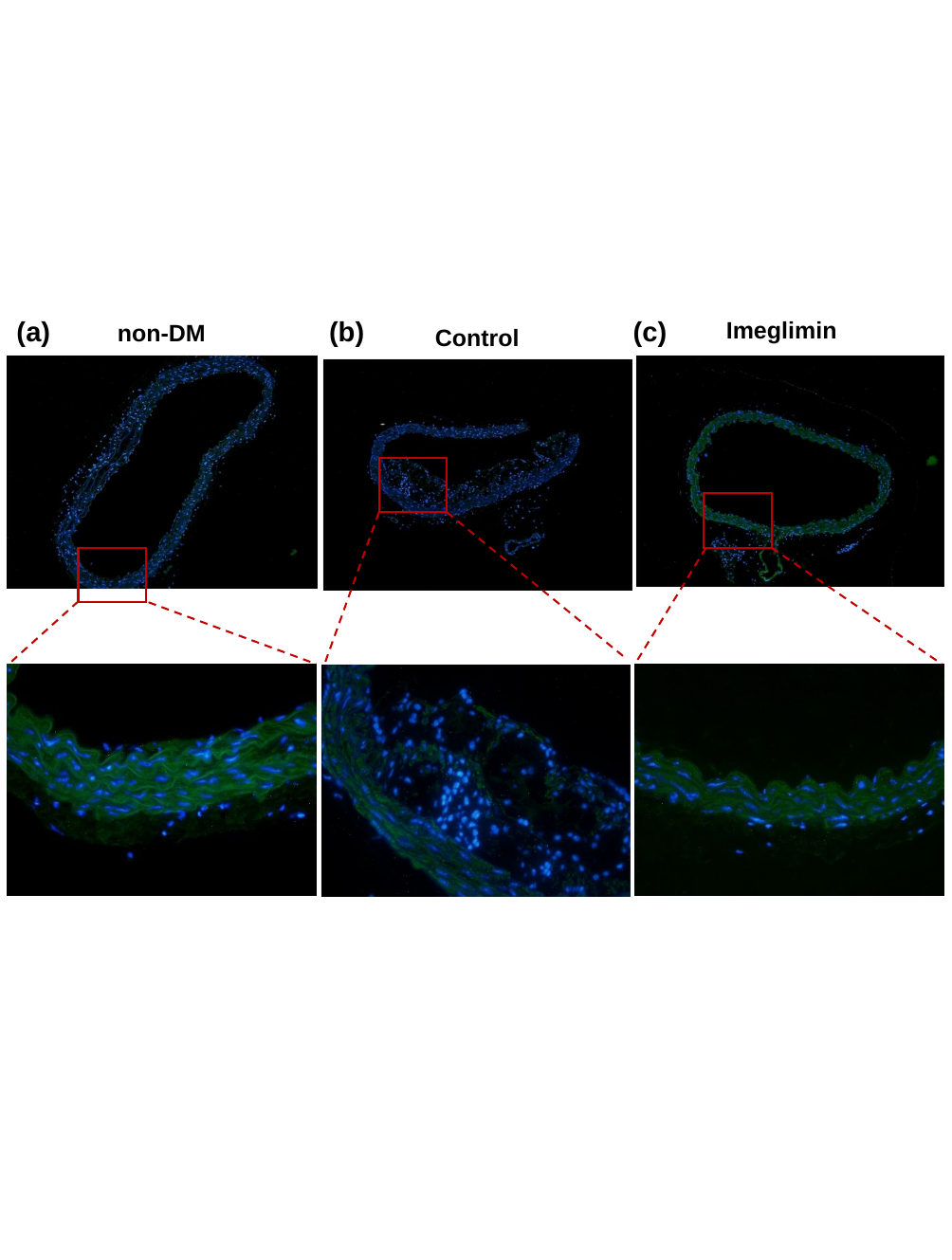

(b)
(c)
(a)
Imeglimin
non-DM
Control

Supplement: Supplementary file 1 — Additional file 1: Figure S1. (a-b) There was no significant difference between untreated DM mice (control group) and imeglimin-treated DM mice (imeglimin group) in food intake and organ weights. Black squares and bars, untreated DM mice (control group); White squares and bars, imeglimin-treated DM mice. n = 9–12. Figure S2. (a)-(c) In α-SMA staining of the thoracic aorta, proliferation and extension of vascular smooth muscle cells into the intima was observed in untreated DM mice (control group) compared non-DM mice. In imeglimin-treated DM mice, plaque development was reduced compared to control group. [file 12933_2024_2189_MOESM1_ESM.pptx]
